# The Efficacy and Tolerability of Prostaglandin Analogues in Treating Systemic Sclerosis-Associated Raynaud Phenomenon: A Systematic Review and Meta-Analysis

**DOI:** 10.1155/ijr/1682081

**Published:** 2024-12-23

**Authors:** Hana Alahmari, Hila Jazayeri, Sindhu R. Johnson

**Affiliations:** ^1^Division of Rheumatology, Department of Medicine, King Khalid University, Abha, Saudi Arabia; ^2^Toronto Scleroderma Program, Schroeder Arthritis Institute, Toronto Western Hospital, Toronto, Ontario, Canada; ^3^Institution of Health Policy, Management, and Evaluation, University of Toronto Canada, Toronto, Ontario, Canada

**Keywords:** prostacyclin analogue, prostaglandins, Raynaud's phenomenon, scleroderma, systemic sclerosis

## Abstract

**Background:** Systemic sclerosis-associated Raynaud phenomenon (SSc-RP) confers poor outcomes, including ulceration, gangrene, autoamputation, and hand disability. Prostaglandin analogues (PG) are a group of prostacyclin-derived drugs with properties that may address underlying complex mechanisms of SSc-RP. This systematic review and meta-analysis evaluated the efficacy and tolerability of PGs in SSc-RP.

**Methods:** We systematically reviewed randomized control trials (RCTs) evaluating PG use in SSc-RP. The primary outcome was the severity of RP attacks. The secondary outcomes were the frequency and duration of RP attacks, healing of digital ulcers, development of new digital ulcers, change of capillary blood flow, patient health-reported outcome measure (PROM-VAS), and tolerability.

**Results:** Eleven RCTs were included, reporting a total of *n* = 1081 individuals with SSc. PG confers a beneficial effect on RP severity in the short-term, weighted Mean difference (WMD) −0.63 (95% CI −0.99, −0.27, *I*^2^ 0%), with no difference in tolerability compared to placebo OR 1.88 (95% CI 1.00, 3.55, *I*^2^ = 1%). PG has nonsignificant trends towards improvement in RP frequency WMD of −0.32 (95% CI −0.76, 0.13, *I*^2^ = 0%), RP duration WMD −4.78 (95% CI −14.69, 5.14, *I*^2^ = 1%), PROM-VAS WMD −4.81 (95% CI −11.31, 1.69, *I*^2^ = 67%), and new or recurrent digital ulcers OR 0.92 (95% CI 0.48, 1.76, *I*^2^ = 34%).

**Conclusion:** PGs are beneficial in the short term to reduce the RP severity and are tolerable. Larger, adequately powered trials are needed for higher certainty evidence across SSc-RP outcomes.

**Summary**

• In this meta-analysis, we demonstrate that prostaglandin analogues effectively control the symptoms of Raynaud phenomenon among people with systemic sclerosis.
• We also found that daily oral prostaglandin reduces the odds of new ulcers by about 10% in the long term.
• Given the aggressiveness and the burden of systemic sclerosis associated with the Raynaud phenomenon on the quality of life, we believe that the finding presented will be of interest to the patients and rheumatologists.

## 1. Introduction

Systemic sclerosis (SSc) is a complex systemic autoimmune rheumatic disease characterized by myofibroblast proliferation, inflammatory vasculopathy, and autoantibody formation [1]. It is a rare disease with a global prevalence of 18.87 per 100,000 individuals [2], predominantly in females between 30 and 50 [2, 3]. Raynaud's phenomenon (RP) is a nearly universal clinical manifestation in about 95% of patients with limited and diffuse subtypes [4, 5]. RP is defined subjectively as at least a biphasic digital color change with pallor (well-demarcated whitening of acral skin), cyanosis (dusky blueness which disappears on rewarming), or suffusion (well-demarcated redness) [6]. Objectively, RP-related SSc can be distinguished from the other underlying pathologies by a unique nail fold capillaroscopy pattern, including giant capillaries and capillary dropout [6]. RP can precede the clinical onset of the illness by several years. It can progress aggressively to digital ischemia associated with severe pain, ulcers, and gangrene in about 1.5%–9% [7], resulting in autoamputation and hand disability [5]. The proposed pathophysiology of RP is a composite of multiple pathways, including endothelial injury with subsequent platelet aggregation, the imbalance between vasoconstrictor and vasodilator molecules, and an increase in radical oxygen species release [8, 9]. Autoantibody–mediated endothelial receptor activation may also contribute to endothelial injury and proinflammatory cell cascade [5]. SSc-RP is distinctively related to fibrointimal hyperplasia due to excessive myofibroblast production and impaired fibrinolysis.

Prostaglandin analogues (PG) may have the ability to halt the multidirectional pathophysiology of RP-SSc [10]. PGs available on the market include iloprost, alprostadil, epoprostenol, beraprost, treprostinil, cisaprost, and selexipag, a prostacyclin receptor agonist. PGs have shared mechanisms of action that address the SSc-RP vasculopathy's complexity, such as platelet antiadhesiveness effects, profibrinolytic activity, antioxidant properties, inhibition of proliferation of medial smooth muscle cells, inhibition of chemotaxis and activation of white cells, reduction of endothelial permeability and inhibition of the vasoconstrictive activity of thromboxane A2, serotonin, leukotrienes, and endothelin [11–13].

Data from observational studies suggest a beneficial effect of PG in ameliorating RP attacks and minimizing the development of digital ulceration [14–17]. A meta-analysis by Pope et al. Two thousand included seven randomized controlled trials (RCTs) from 1988 to 1996, which concluded an unimpressive efficacy of iloprost and cisaprost [18]. However, this meta-analysis was conducted over two decades ago, and only two PGs were included. Another meta-analysis by Lustig et al. focused on iloprost efficacy in SSc-RP and concluded that iloprost might lead to little or no difference in the frequency or severity [19]. There is also a knowledge gap about PG's effect on quality of life and functional measures. We aim to evaluate the efficacy and tolerability of PG in SSc-RP.

## 2. Methods

This study complies with the Preferred Reporting Item for Systematic Review and Meta-analysis Protocols (PRISMA-S 2020) standards [1].

### 2.1. Eligibility Criteria

We included all RCTs in which at least one PG was compared to a placebo or usual care medication from database inception to May 20, 2024. Blinded, nonblinded, parallel, or crossover trials were considered. Three arm studies (for example, comparing two doses and one comparator as a third group) were included. Studies that reported mixed populations of patients with diagnoses other than SSc were included if the SSc subgroup was analysed independently for the outcome of interest. There were no restrictions on language or year of publication for the inclusion.

### 2.2. Subjects

Trials that included adult (≥ 18 years) participants classified with SSc according to the 2013 American College of Rheumatology (ACR)/European Alliance of Associations for Rheumatology (EULAR) classification criteria [20] or 1980 ACR criteria for SSc (for trials published before the establishment of 2013 criteria) were included. RP could be present in the fingers, toes, or both. An ulcer was defined as an epithelial loss with discernible depth secondary to vasospasm and hypoperfusion [21–24].

### 2.3. Intervention

Eligible studies were those that administered one of the following PG medications: iloprost, alprostadil, epoprostenol, beraprost, treprostinil, cisaprost, and selexipag. Studies reported oral or intravenous (IV) administration were included. Studies using vasodilators concomitantly with PG for other reasons (e.g., calcium channel blocker for hypertension or phosphodiesterase for pulmonary hypertension) were not excluded. The comparators were at least one arm of placebo or other intervention. We excluded the studies on the effect of topical PG or whether the participants with primary RP were included without an independent subgroup analysis of SSc subjects.

### 2.4. Literature Search

With the assistance of the librarian information specialist (M.A.A.) and two reviewers (H.A. and H.J.), we ran searches of OVID MEDLINE, Embase, and the Cochrane Controlled Trials Register from the database inception to May 20, 2024. The keywords used as terms or subheadings were *Prostaglandin*, *Prostacyclin*, *PGI2*, *PGE2*, *Iloprost*, *Alprostadil*, *Epoprostenol*, *Beraprost*, *Cisaprost*, *Treprostinil*, *Selexipag*, *Raynaud's phenomenon*, *Raynaud's disease*, *Vasospasm*, *Systemic sclerosis*, *Diffuse systemic sclerosis*, *Limited systemic sclerosis*, *Scleroderma*, *CREST*, and *Connective tissue disease.* For a comprehensive search, we search by the medication's brand names. As described in Table 1 (OVID MEDLINE search strategy), truncation and adjacency tools were used. Using the term “systemic sclerosis,” ClinicalTrials.gov trial and the ACR conference abstracts for unpublished studies were searched. The reference lists of the eligible studies were manually reviewed for relevant studies.

### 2.5. Data Management

All citations were imported to the reference manager (EndNote 20). Duplicate citations were removed. A standardized template was used for data abstraction. Pilot testing of the data abstraction template was conducted for possible modification. Two reviewers independently screened the title and abstract of each citation for full-text review. Non-English articles were translated using Microsoft translator software and a native speaker interpreter. All disagreements between the two reviewers were discussed with the third reviewer (S.R.J.).

### 2.6. Study Characteristics

Study authors, title, design, year of publication, funding, number of patients in each arm, demographic and clinical characteristics, outcomes of interest, follow-up time, number of missing participants, and adverse events reported were collected.

### 2.7. Outcomes

The primary outcome was the change from the baseline in the severity of RP attacks measured on validated scales between 4 and 6 weeks of the intervention.

Secondary outcomes included the frequency of RP attacks, duration of RP attack in minutes, healing of ulcers secondary to RP, development of new acral ulcers secondary to RP, change in the capillary blood flow posttreatment (measured by Doppler ultrasound or laser Doppler imaging/laser Doppler flowmetry), and change in the patient's health-related outcome. Tolerability was measured by the number of withdrawals from the trial due to adverse events. Outcome assessment was categorized as short-term (< 4 months) and long-term (≥ 4 months). If the acral ulcer was referred to as any lesion (fissures, skin peeling) or if the presence of an ulcer was indeterminant, the result was not included in this study. Each outcome with the corresponding measurement is summarized in Table 2.

### 2.8. Risk of Bias

One reviewer assessed the risk of bias using the Cochrane Risk of Bias 2 tool (ROB2) [25]. Trial protocols were reviewed where available.

### 2.9. Data Synthesis

Meta-analysis was done using RevMan software (Version 5.4.1) [26]. The mean difference (MD) was used for continuous outcomes to synthesize the effect estimate, whereas the odds ratio (OR) was used for the dichotomous outcomes. We used a random effects model for the included studies in each outcome eligible for meta-analysis. We performed the meta-analyses using the weighted mean differences (WMDs) with a 95% confidence interval (95% CI) for the continuous outcomes. We used a log OR for a pooled estimate of proportional outcomes. In a three-arm study where the drug was given in two doses over the trial, we calculated the average of the estimated effect. The weighted study's point of estimate and the weighted overall estimate effect are described in a forest plot for each outcome. We have placed a value of 0.5 when there is a “zero” event. The heterogeneity statistics tested by *I*^2^ statistics yield a percentage of total variation across studies due to heterogeneity beyond the random effect (0%: no heterogeneity; 25%: low heterogeneity; 50%: moderate heterogeneity; 75%: high heterogeneity) [27]. The variance between studies was estimated using the tau^2^ metrics.

We evaluated the certainty of the evidence using the GRADE (Grading of Recommendations, Assessment, Development, and Evaluations) methodology [28]. Evidence from RCTs was classified as having high certainty but can be downgraded for risk of bias, imprecision, inconsistency, indirectness, and publication bias.

## 3. Results

### 3.1. Search Results

**Figure 1:**
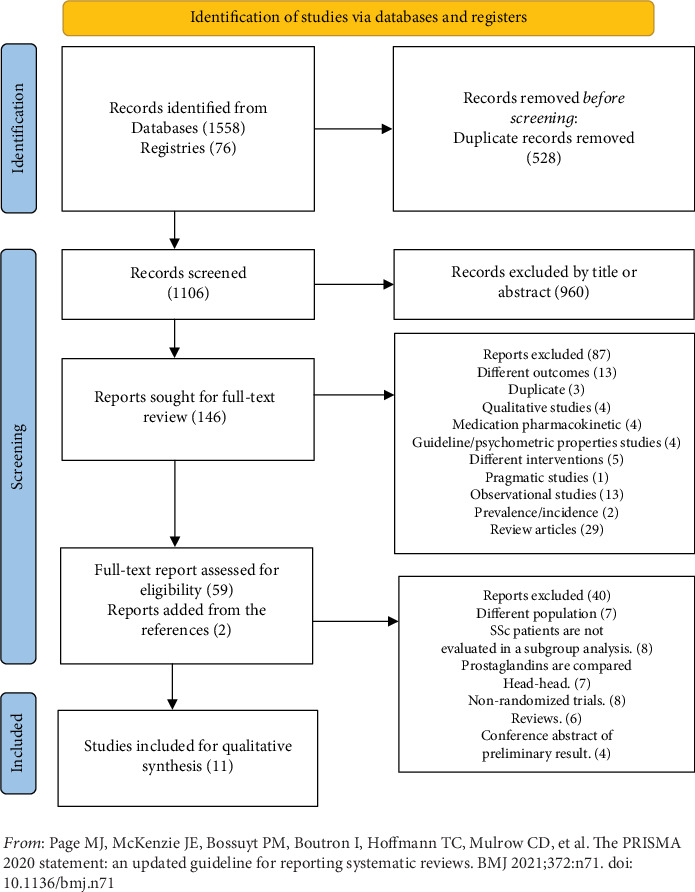
Preferred Reporting Item for Systematic Review and Meta-Analysis (PRISMA) flow diagram.

Of the 1634 studies identified, 61 had a full-text review, and 50 were excluded due to the type of the included participants (mixed with other rheumatic disease patients or primary RP) or the comparator being another PG (head-to-head trials). The search strategy is outlined in Table 1. The PRISMA flow diagram is illustrated in Figure 1.

Our search yielded 11 RCTs [29–39], of which 10 were parallel, and 1 was crossover designs. The studies were published between 1981 and 2017 and included 5 PG classes (*n* = 6 iloprost, *n* = 2 PGE1, *n* = 1 beraprost, *n* = 1 Treprostinil, and *n* = 1 selexipag), often against placebo. All trials contained only patients with SSc, except one where the SSc subjects had extractable data. Study sample sizes ranged from 23 to 308 patients, with a total of 1081 patients included. The time points of outcome evaluation range from 2 weeks of treatment to 1 year. Most of the studies are intended to be conducted over the winter. The characteristics of the included studies are summarized in Table 3.

### 3.2. Outcomes Summary

Table 4 summarizes the study outcomes, and Table 5 presents a meta-analysis.

### 3.3. RP Severity

**Figure 2:**
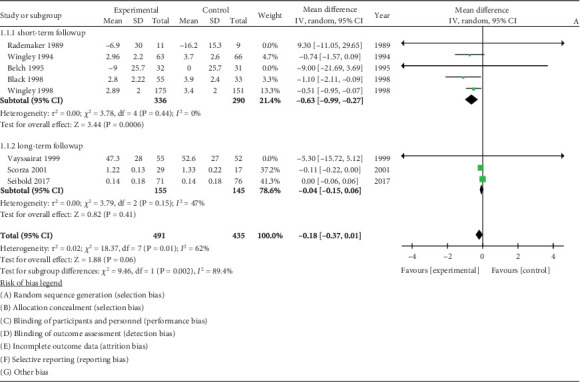
Forest plot of prostaglandin (PG) versus control effect on Raynaud phenomenon severity.

Nine of 10 studies were included in the analysis. The study by Martin et al. [35] was omitted because the carryover effect could not be excluded. Iloprost significantly reduced RP severity in the short term with a WMD −0.63 (95% CI −0.99, −0.27, *I*^2^ = 47%).  Figure 2. The long-term effect was sustained but not statistically significant WMD −0.18 (95% CI −0.38, 0.01).

### 3.4. RP Frequency

**Figure 3:**
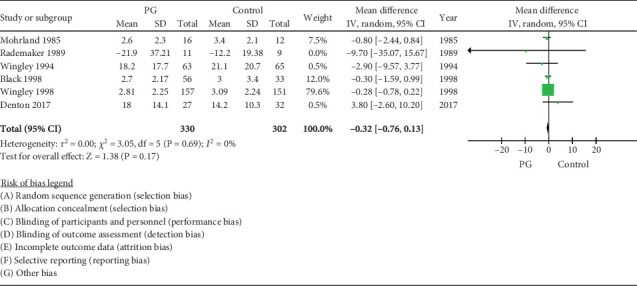
Forest plot of prostaglandin (PG) versus control effect on Raynaud phenomenon frequency.

Six RCTs with a total sample size of 330 were meta-analysed. The frequency of attacks in the PG versus placebo group was not different, with a WMD of −0.32 (95% CI −0.76, 0.13, *I*^2^ = 0%) (Figure 3).

### 3.5. RP Duration

**Figure 4:**
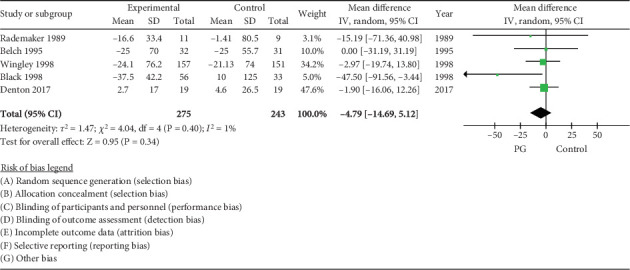
Forest plot of prostaglandin (PG) versus control effect on Raynaud phenomenon duration.

The pooled estimate of effect from five RCTs showed no significant change in the duration of the attacks between treatment and placebo, WMD −4.78 (95% CI −14.69, 5.14, *I*^2^ = 1%) (Figure 4).

### 3.6. PROM-VAS

**Figure 5:**
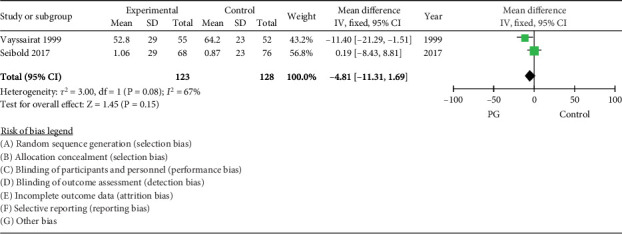
Forest plot of prostaglandin (PG) versus control effect on patient reported outcome using visual analogue scale.

The pooled estimate from two trials did not demonstrate a significant effect of PG on PROM-VAS in the long term, WMD −4.81 (95% CI −11.31, 1.69, *I*^2^ = 67%) (Figure 5).

### 3.7. Digital Ulcer Healing

**Figure 6:**
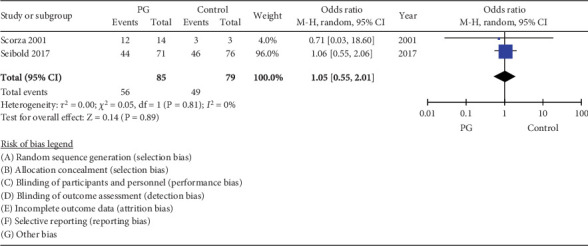
Forest plot of prostaglandin (PG) versus control effect on digital ulcer healing in long-term follow-up (≥ 4 months).

The pooled estimate from 2 RCTs of 164 participants did not demonstrate a significant effect of PG on ulcer healing in the long term with an overall OR of 1.05 (95% CI 0.55, 2.01, *I*^2^ = 0%) (Figure 6).

### 3.8. New or Recurrent Digital Ulcers

**Figure 7:**
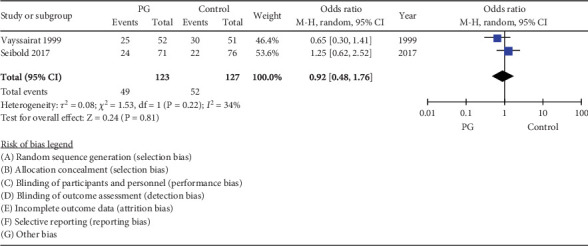
Forest plot of prostaglandin (PG) versus control effect on new or recurrent digital ulcers in long-term follow-up (≥ 4 months).

The pooled estimate from 2 RCTs of 250 participants did not demonstrate a significant effect of PG on developing new or recurrent ulcers OR 0.92 (95% CI 0.48, 1.76, *I*^2^ = 34%) (Figure 7).

### 3.9. Intolerability

**Figure 8:**
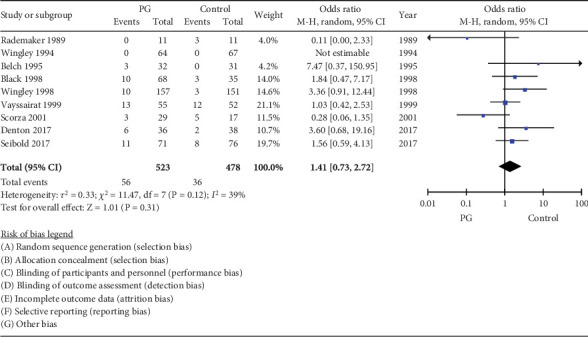
Forest plot of PG withdrawal due to intolerability compared to the control group.

Ninety-two of 1001 (9.19%) subjects (*n* = 56 PG arm; *n* = 36 control arm) withdrew from the trials due to adverse events (10.7% vs. 7.3%), respectively. Drug withdrawal was considered a surrogate measure of drug intolerability if the reason of withdrawal was not explained by the author. There was no significant difference in tolerability between PG and control medications with OR 1.41 (95% CI 0.73–2.72, *I*^2^ = 39%) (Figure 8).

### 3.10. Certainty of Evidence

The certainty of the evidence for the beneficial effect of PG on the frequency of RP attacks was moderate. The certainty of evidence for the severity and duration of RP by PG was low (Table 6).

### 3.11. Risk of Bias

**Figure 9:**
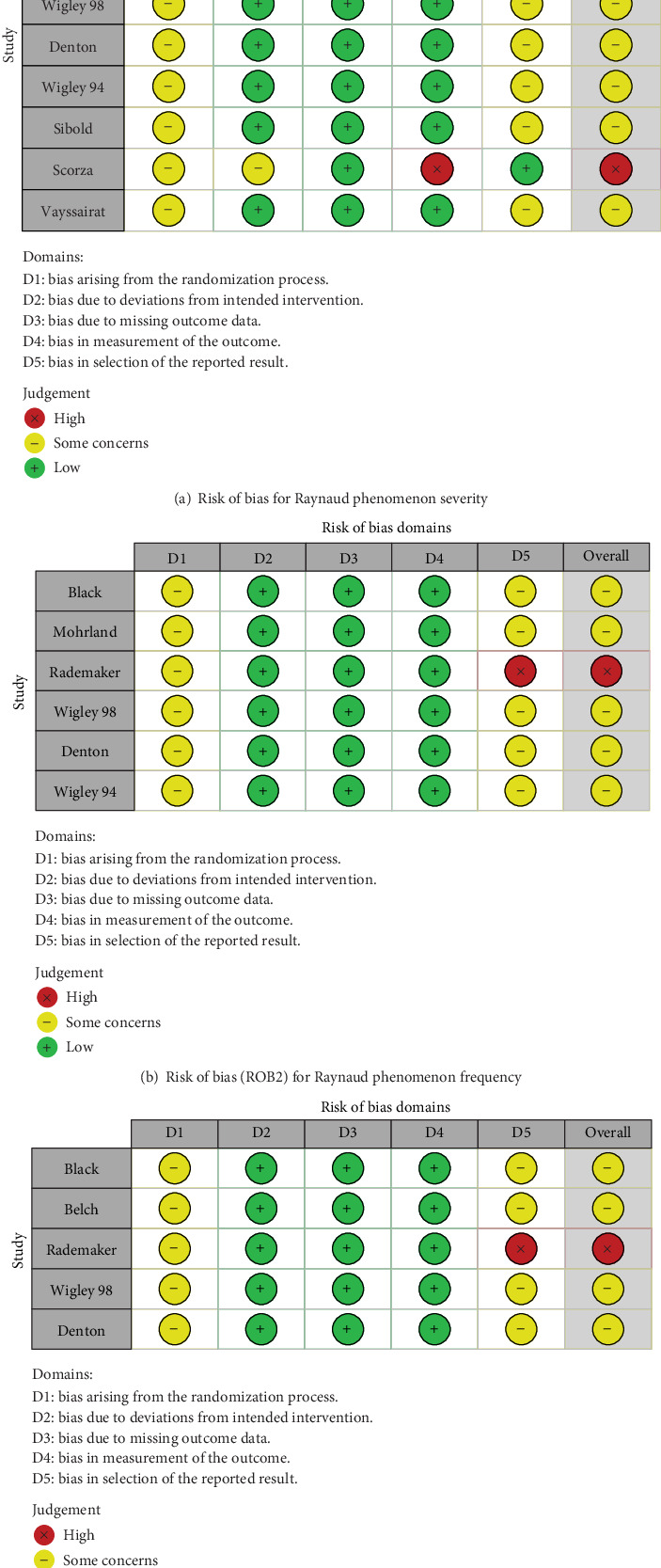
Risk of bias traffic light plots for Raynaud phenomenon-related outcomes.

Traffic light plots summarize each domain's rating of the risk of bias (Figures 9(a), 9(b), and 9(c)). Some studies could not clearly distinguish the number of patients included in the final analysis from the number of patients initially randomized, and we did not downgrade the quality rating of these trials. Of 11 studies, protocols were only found for three studies. All studies have been downgraded in D1 and D5 because of information ambiguity in the sequence concealment. There is a high risk of bias in the study by Rademaker et al. due to multiple outcome analyses.

## 4. Discussion

Our evidence synthesis suggests that PG may help individuals with SSc-RP. We demonstrate that PG conferred a significant beneficial effect on the severity of pain related to RP in the short term and did not demonstrate worse tolerability compared to comparators. While not statistically significant, PG showed a potential improvement in the frequency and duration of RP. The evidence of their efficacy on patient-reported outcomes and digital ulcer–related outcomes was inconclusive.

Our findings contribute to a field of conflicting conclusions. One network meta-analysis that evaluated all the pharmacological treatments used for secondary RP concluded that IV PG was not superior to placebo [40]. Pope et al. [5] concluded that iloprost and cisaprost effectively decreased the number of RP attacks. Differences in the methodological approaches across studies can explain this discrepancy, such as reporting posttreatment data versus the change from baseline or the percentage change from the baseline. Applying different criteria for the inclusion of study data is an additional explanation.

High heterogeneity among the scales and scores used to record the severity of attacks and a significant difference in the assessment time are major factors that explain the borderline pooled estimate of the severity outcome. This is supported by the improved precision of the estimate when the studies stratified based on the time of outcome assessment to short-term and long-term effects. The length of long-term follow-up varied, and if iloprost has a delayed onset of action, then trials of insufficient follow-up time may be falsely negative even if the effect is helpful later. Most of the studies were conducted during winter to eliminate seasonal variability as a confounder.

The change in the capillary flow in response to the treatment was only addressed in two studies. Using laser Doppler flowmetry output, Rademaker et al. demonstrated statistically significant increased blood flow from the baseline with iloprost at Weeks 4 and 8 compared to nifedipine. This effect declined and was no longer evident at Week 16. This might emphasize the beneficial impact of cyclical iloprost rather than a 3-day infusion. Martin et al. also showed short-term improvement in capillary blood flow using quantified infrared thermography, a surrogate measure of changes in peripheral blood flow [41].

The available evidence is insufficient to draw firm conclusions about digital ulcer outcomes. However, Vayssairat et al. showed that 6 months of daily beraprost significantly reduced the burden of new ulcer development and delayed the evolution of new ulcers [26]. A similar outcome was observed with treatment with daily oral treprostinil for 5 months in individuals with digital ulcers [29]. The same study illustrated a trend of continuous improvement in net ulcer burden, although it was not statistically significant.

We found similarly low number of withdrawals in the PG treatment group compared to the control groups taking placebo or another vasodilator indicating tolerability. A similar conclusion was drawn from the network meta-analysis of all medications used for secondary RP, although the tolerability criteria were broader than those in our study [36]. The most commonly reported symptoms were postural hypotension, flushing, and headache.

Strengths of this study are the systematic methodology of our review, meta-analysis of a broad range of efficacy outcomes deemed clinically important in SSc-RP, and our tolerability evaluation, as this can be a critical trade-off. Compared to other studies, our meta-analysis is narrow in scope, to only individuals with SSc in controlled trials. Limitations of this study include our inability to evaluate all PG classes, the small number of included trials, the low certainty of evidence, and the small sample sizes. Our demonstration of treatment effects in a similar direction across several outcome measures suggests that the lack of statistical significance is likely attributable to low power from the low sample sizes and not a demonstration of a lack of beneficial effect. Some pooled effects were estimated from meta-analyses of only two studies which carries a risk of substantial uncertainty of the effect estimate. Another limitation is the heterogeneity of the measures related to efficacy outcomes, which might impact the robustness of the results. Regarding publication bias, it was not evaluated due to too few studies. However, publication bias cannot be excluded as the negative study is less likely to be published.

In summary, PG can ameliorate the severity of SSc-RP in the short term. If given in a cyclic, regular pattern, they may confer additional beneficial effects at the individual level. Our work suggests that larger, adequately powered trials are needed to generate high-certainty evidence.

## Figures and Tables

**Table 1:** Search strategy.

	**Query**	**Results from May 20, 2024**	**Notes**
1	(Prostacyclin or prostaglandin or prostanoid or PGI2 or PGE2).mp. [mp=title, book title, abstract, original title, name of substance word, subject heading word, floating sub-heading word, keyword heading word, organism supplementary concept word, protocol supplementary concept word, rare disease supplementary concept word, unique identifier, synonyms]	120,026	1–13 drug terms and keywords. Some drugs have keywords only.
2	exp Receptors, Epoprostenol/or exp Epoprostenol/	13,145	
3	exp Iloprost/	2121	
4	Treprostinil.mp.	731	
5	Cisaprost. mp.	1	
6	Alprostadil.mp. or exp Alprostadil/	7457	
7	Beraprost.mp.	547	
8	Selexipag.mp.	239	
9	prostacyclin. mp.	14,710	
10	prostaglandin.mp.	100,473	
11	prostanoid.mp.	5769	
12	PGI2.mp.	6815	
13	PGE2.mp.	27,927	
14	Flolan. mp.	59	From 14-19 are the brand names
15	Ventavis.mp.	14	
16	Remodulin. mp.	23	
17	(Caverjet or Edex, or Prostin VR).mp. [mp=title, book title, abstract, original title, name of substance word, subject heading word, floating sub-heading word, keyword heading word, organism supplementary concept word, protocol supplementary concept word, rare disease supplementary concept word, unique identifier, synonyms]	32	
18	(Dorner or Yamanouchi or Kaken).mp. [mp = title, book title, abstract, original title, name of substance word, subject heading word, floating sub-heading word, keyword heading word, organism supplementary concept word, protocol supplementary concept word, rare disease supplementary concept word, unique identifier, synonyms]	136	
19	UPTRAVI.mp.	14	
20	1 or 2 or 3 or 4 or 5 or 6 or 7 or 8 or 9 or 10 or 11 or 12 or 13 or 14 or 15 or 16 or 17 or 18 or 19	125,799	All prostaglandin drugs
21	exp Raynaud Disease/or Raynaud⁣^∗^.mp.	10,291	
22	(Raynaud's phenomenon⁣^∗^ or Raynaud's syndrome).mp. [mp=title, book title, abstract, original title, name of substance word, subject heading word, floating sub-heading word, keyword heading word, organism supplementary concept word, protocol supplementary concept word, rare disease supplementary concept word, unique identifier, synonyms]	5714	
23	Vasospasm⁣^∗^.mp.	17,059	
24	21 or 22 or 23	26,937	All of Raynaud's phenomenon Mesh & Keywords
25	exp Scleroderma, Systemic/or exp Scleroderma, Localized/	25,717	
26	Systemic sclerosis. mp.	17,943	
27	exp CREST Syndrome/or CREST.mp.	30,221	
28	connective tissue diseases/or lupus erythematosus, cutaneous/or lupus erythematosus, systemic/or rheumatic diseases/or scleroderma, localized/or scleroderma, systemic/or undifferentiated connective tissue diseases/	114,119	
29	25 or 26 or 27 or 28	148,298	All systemic sclerosis Mesh and keywords
30	20 and 24 and 29	224	The final search connects the three constructs using (AND) articulation
31	#24.mp. adj2 (exp Scleroderma, Systemic/or exp Scleroderma, Localized/or Systemic sclerosis. mp. or (exp CREST Syndrome/or CREST.mp.) or (connective tissue diseases/or lupus erythematosus, cutaneous/or lupus erythematosus, systemic/or rheumatic diseases/or scleroderma, localized/or scleroderma, systemic/or undifferentiated connective tissue diseases/)) [mp = title, book title, abstract, original title, name of substance word, subject heading word, floating sub-heading word, keyword heading word, organism supplementary concept word, protocol supplementary concept word, rare disease supplementary concept word, unique identifier, synonyms]	1142	Using adjacency between Raynaud's and systemic sclerosis
32	20 and 31	7	Adding adjacency to the final search

**Table 2:** Summary of meta-analysis of efficacy and tolerability of prostaglandin analogues in SSc-RP.

**Outcome**	**Weighted mean difference/odds ratio (95% confidence interval)**	**Heterogeneity ** **I** ^2^
Raynaud's severity		
Short-term	−0.63 (−0.99, −0.27)	0%
Long-term	−0.04 (−0.15, 0.06)	47%
Overall	−0.18 (−0.37, 0.01)	62%
Raynaud's frequency	−0.32 (−0.76, 0.13)	0%
Raynaud's duration	−4.79 (−14.69, 5.12)	1%
PROM (VAS)	−4.81 (−11.31, 1.69)	67%
Digital ulcer healing (long-term)	1.05 (0.55, 2.01)	0%
New or recurrent digital ulcers (long-term)	0.92 (0.48, 1.76)	34%
Withdrawal due to intolerability	1.41 (0.73, 2.72)	39%

Abbreviations: PROM, patient-reported outcomes; VAS, Visual Analogue Scale.

**Table 3:** Summary of the study characteristics.

**Author/year**	**Trial design**	**Season**	**PG/control**	**Dose/duration**	**Number of participants**	**DU/DI**	**Follow-up period (weeks)**
Martin et al. (1981) [39]	Crossover with washout Period 4–5 wks SSc	N/A	PGE1/placeboI V	From 6 up to 10 ng/kg/min (3 days)	24	DU 41.60% vs. DI 26%	2
Mohrland et al. (1985) [38]	Parallel and double-blind SSc	Winter	PG E1/placebo IV	10 ng/kg/min (3 days)	31	DU 14% vs. 35.70%	2 and 4
Rademaker et al. (1989) [37]	Parallel, double-blind SSc, and primary RP	Winter	Iloprost/nifedipine IV	From 5 to 2.0 ng/kg/min (3 days and another single dose at 8 wks)	23	DU 3.5 vs. 4.3	4, 8, 12, and 16
Wigley et al. (1994) [36]	Parallel and double-blind SSc	Winter	Iloprost/placebo IV	0.5 to max. 2.0 ng/kg/min (5 days)	131	DU 9.33 ± 7.87 vs. 9.12 ± 7.61	3, 6, and 9
Wigley et al. (1998) [33]	Parallel and double-blind SSc	Winter	Iloprost/placebo PO	50 *μ*g BID (6 wks)	308	DU 21% vs. 20.50%	6
Black et al. (1998) [34]	Parallel and double-blind SSc	Winter	Iloprost/placebo PO	50 *μ*g to max.100 *μ*g BID (6 wks)	103	DU 26% in each group	6–12
Vayssairat (1999) [30]	Parallel and double-blind SSc	Two periods (spring/summer and winter)	Beraprost/placebo PO	60 *μ*g TID (6 months)	107	DU 96% vs. 93%DI 100%	24
Belch et al. (1995) [35]	Parallel and double-blind SSc	Winter	Iloprost/placebo PO	50 *μ*g to max. 150 *μ*g. BID (10 days)	63	N/A	2
Scorza et al. (2001) [32]	Parallel and single blind SSc	Over 1 year	Iloprost/nifidipine IV	0.1 to a maximum of 2.0 ng/kg/*μ*g (5 days every 6 wks for 12 months)	46	DU 48.2% vs. 17.6%	24–52
Seibold et al. (2017) [29]	Parallel and double-blind, open-label SSc	N/A	Treprostinil diethanolamine/placebo PO	0.25 mg to max. 16 mg BID (20 wks)	147	DU 100% of each group	20
Denton et al. (2017) [31]	Parallel and double-blind SSc	Yes	Selexipag/placebo PO	200*–*1600 *μ*g BID (8 wks)	74	DU 11.10% vs. 18.40%	8

Abbreviations: DI, digital ischemia; DU, digital ulcer; PG, prostaglandin; PGE1, prostaglandin E1; Wks, weeks.

**Table 4:** Summary of the studies' endpoints.

**Outcomes**	**Study**	**Treatment**	**Outcome measures**	**Time points**	**Effect estimates. PG/placebo (SD)**
RP severity	Martin et al. (1981) [39]	PGE1 IV	RP VAS	2 weeks	2.19 cm vs. 0.91 cm improvement from the baseline
	Rademaker et al. (1989) [37]	Iloprost IV	Diary	8 weeks	−6.9 (30) vs. −16.2 (15.3)^¥^
	Wigley et al. (1994) [36]	Iloprost IV	RP VAS	6 weeks	2.96 (2.2) vs. 3.67 (2.6)^a^
	Wigley et al. (1998) [33]	Iloprost PO	RCS	6 weeks	2.89 (2) vs. 3.4 (2.1)^a^
	Black et al. (1998) [34]	Iloprost PO	RCS	6 weeks	2.8 (2.2) vs. 3.9 (2.4)^a^
	Vayssairat (1999) [30]	Beraprost PO	RP VAS	24 weeks	47.3 (28) vs. 52.6 (27)^a^
	Belch et al. (1995) [35]	Iloprost PO	0 = none; 1 = mild; 2 = moderate; 3 = severe	2 weeks	−9 (25.7) vs. 0 (25.7)^¶^
	Scorza et al. (2001) [32]	Iloprost IV	RP severity score	24 weeks	1.22 (0.13) vs. 1.33 (0.22)^¶^
	Seibold et al. (2017) [29]	Treprostinil PO	RP severity score	20 weeks	0.14 (0.19) vs. 0.14 (0.19)^a^
	Denton et al. (2017) [31]	Selexipag PO	RCS	8 weeks	There is no significant change from the baseline

RP frequency	Mohrland et al. (1985) [38]	PGE1 IV	Diary per day	4 weeks	2.6 ± (2.3)/3.4 ± (2.1)^a^
	Rademaker et al. (1989) [37]	Iloprost IV	Diary per day	8 weeks	−21.9 (37.2)/-12.2 (19.3)^¥^
	Wigley et al. (1994) [36]	Iloprost IV	Diary per day	6 weeks	18.2 ± (17.7)/21.1 ± (20.7)^a^
	Wigley et al. (1998) [33]	Iloprost PO	Diary per day	6 weeks	2.81 ± (2.25)/3.09 ± (2.24)^a^
	Black et al. (1998) [34]	Iloprost PO	Diary per day	6 weeks	2.7 (2.2)/3 (3.4)^a^
	Denton et al. (2017) [31]	Selexipag PO	Diary per week	8 weeks	18.0 (14.1)/14.2 (10.3)^a^

RP duration	Rademaker et al. (1989) [37]	Iloprost IV	Per min/day	8 weeks	−16.6 (33.4)/−1.41 (80.5)^¥^
	Belch et al. (1995) [35]	Iloprost PO	Per min/week	2 weeks	−25 (70) vs. −25 (55.7)^¥^
	Wigley et al. (1998) [33]	Iloprost PO	Per min/day	6 weeks	−24 (76.2)/ −21.13 (74)^¥^
	Black et al. (1998) [34]	Iloprost PO	Per min/day	6 weeks	−37.5 (42.21) vs. 10 (125)^¥^
	Denton et al. (2017) [31]	Selexipag PO	Per min/week	8 weeks	2.7 (17) vs. 4.6 (26.5)^a^

Capillary blood flow	Martin et al. (1981) [39]	PGE IV	Quantified infrared thermography	2 weeks	1.56°C vs. 0.7°C improvement
	Rademaker et al. (1989) [37]	Iloprost IV	Laser Doppler flowmetry output (mV)	8 weeks	919.4 vs. −140.6^¶^

Patient-reported outcome	Wigley et al. (1994) [36]	Iloprost IV	Ordinal scale [1–4], 1 = great improvement, 0 = worse	9 weeks	0.86 (0.69) vs. 0.78 (0.71)^a^
	Black et al. (1998) [34]	Iloprost PO	PGA	6 weeks	60% vs. 44% improvement
	Vayssairat (1999) [30]	Beraprost PO	VAS	24 weeks	52.8 (29) vs. 64.2 (23)^a^
	Seibold et al. (2017) [29]	Treprostinil PO	VAS HAQ	20 weeks	1.06 vs. 0.87^a^ 0.48 vs. 0.43^a^

DU healing	Martin et al. (1981) [39]	PGE IV	No specific definition	2 weeks	2/5 (40%)
	Rademaker et al. (1989) [37]	Iloprost IV	No specific definition	16 weeks	0.6 (0.3) vs. 1.4 (0.5)
	Wigley et al. (1994) [36]	Iloprost IV	A reduction in the number of finger lesions from the baseline of at least 50% No specific definition	9 weeks	1.4/9.33 (15%) vs. 2.3/9.12 (25%)
	Black et al. (1998) [34]	Iloprost PO	No specific definition	6 weeks	4/15 (26.6%) vs. 1/7 (14%)
	Scorza et al. (2001) [32]	Iloprost IV	Healing of a designated cardinal ulcer	52 weeks	12/14 (85.7) vs. 3/3 (100%)
	Seibold et al. (2017) [29]	Treprostinil PO		20 weeks	44/71 (62%) vs. 46/76 (61%)

New or recurrent DU	Vayssairat (1999) [30]	Beraprost PO	Not mentioned	24 weeks	25/52 (48%) vs. 30/51 (58.8%)
	Seibold et al. (2017) [29]	Iloprost PO	If only one active ulcer was present at entry, it was designated as the cardinal ulcer	20 weeks	24/71 (34%) vs. 22/76 (29%)

Withdrawal due to intolerability	Rademaker et al. (1989) [37]	Iloprost IV		16 weeks	0/11 vs. 3/11
	Wigley et al. (1994) [36]	Iloprost IV		9 weeks	0/64 vs. 0/67
	Wigley et al. (1998) [33]	Iloprost PO		6 weeks	10/157 vs. 3/151
	Black et al. (1998) [34]	Iloprost PO		12 weeks	10/68 vs. 3/35
	Belch et al. (1995) [35]	Iloprost PO		2 weeks	3/32 vs. 0/31
	Scorza et al. (2001) [32]	Iloprost IV		52 weeks	3/29 vs. 5/17
	Vayssairat (1999) [30]	Beraprost PO		24 weeks	13/55 vs. 12/52
	Seibold et al. (2017) [29]	Treprostinil PO		20 weeks	11/71 vs. 8/76
	Denton et al. (2017) [31]	Selexipag PO		8 weeks	6/36 vs. 2/38

Abbreviations: HAQ, Health Assessment Questionnaire; IV, intravenous; Min, minutes; PGA, Physician Global Assessment; PGE, prostaglandin E; PO, per oral; RCS, Raynaud condition score; RP, Raynaud phenomenon; VAS, Visual Analogue Scale.

^a^Posttreatment means.

^¶^Change from the baseline.

^¥^Percentage change from baseline.

**Table 5:** Summary of the outcomes and the corresponding measurements.

**Outcome**	**Outcome measure**
The change from the baseline in the frequency of RP attacks at 6 weeks of the intervention	The frequency of the attacks is obtained from the patient's diary.
The severity of RP attacks	RCS or VAS or Likert scales
Duration of RP attack in minutes.	Patient dairy
Healing of ongoing acral ulcers secondary to RP	Physician assessment
Development of new acral ulcers secondary to RP	Physician assessment
Change in the capillary blood flow post-treatment	Measured by Doppler ultrasound or laser Doppler ultrasound imaging/laser Doppler flowmetry
Change in the patient's health-related outcome.	Pt. GA, PGA, HAQ-DI, SF36, or other validated scales.
Intolerability	Withdrawal proportion due to the drug AE.

Abbreviations: AE, adverse events; HAQ-DI, Health Assessment Questionnaire-Disability Index; PGA, Physician Global Assessment; Pt GA, Patient Global Assessment; RCS, Raynaud condition score; RP, Raynaud phenomenon; SF36, Short Form Health Survey; VAS, Visual Analogue Scale.

**Table 6:** Evaluation of certainty of evidence using Grading of Recommendations, Assessment, Development, and Evaluations (GRADE).

**Certainty assessment**							**No. of patients**		**Effect**		**Certainty**
**No. of studies**	**Study design**	**Risk of bias**	**Inconsistency**	**Indirectness**	**Imprecision**	**Other considerations**	**PG**	**Placebo**	**Relative (95% CI)**	**Absolute (95% CI)**	
RP Severity											
8	Randomized trials	Very serious^a^	Not serious^b^	Not serious	Not serious	None	491	435	—	MD 0.18 lower (0.37 lower to 0.01 higher)	⨁⨁◯◯ Low^a^^,^^b^
RP Frequency											
6	Randomized trials	Serious^c^	Not serious	Not serious	Not serious	None	329	302	—	MD 0.32 lower (0.76 lower to 0.13 higher)	⨁⨁⨁◯ Moderate^c^
RP Duration											
5	Randomized trials	Serious^c^	Not serious	Not serious	Serious^d^	None	275	243	—	MD 4.79 lower (14.69 lower to 5.12 higher)	⨁⨁◯◯ Low^c^^,^^d^

Abbreviations: CI, confidence interval; MD, mean difference.

^a^Two studies with high risk of bias.

^b^Moderate heterogeneity.

^c^Overall allocation concerns and high risk of selective reporting.

^d^Wide confidence interval.

## Data Availability

Data is available on request from the authors.
